# IL-17A in Psoriasis and Beyond: Cardiovascular and Metabolic Implications

**DOI:** 10.3389/fimmu.2019.03096

**Published:** 2020-01-15

**Authors:** Esther von Stebut, Wolf-Henning Boehncke, Kamran Ghoreschi, Tommaso Gori, Ziya Kaya, Diamant Thaci, Andreas Schäffler

**Affiliations:** ^1^Department of Dermatology, University of Cologne, Cologne, Germany; ^2^Division of Dermatology and Venereology, Geneva University Hospitals, Geneva, Switzerland; ^3^Department of Pathology and Immunology, Faculty of Medicine, University de Geneva, Geneva, Switzerland; ^4^Department of Dermatology, Venereology and Allergology, Charité—Universitätsmedizin Berlin, Berlin, Germany; ^5^Center of Cardiology—Cardiology I, University Medical Center Mainz, Mainz, Germany; ^6^German Center for Cardiovascular Research, University Center Mainz, Mainz, Germany; ^7^Department of Internal Medicine III, University of Heidelberg, Heidelberg, Germany; ^8^Institute and Comprehensive Center of Inflammation Medicine, University of Lübeck, Lübeck, Germany; ^9^Department of Internal Medicine III, Giessen University Hospital, Giessen, Germany

**Keywords:** IL-17A, psoriasis, comorbidities, cardiovascular, diabetes

## Abstract

Interleukin 17A (IL-17A) is one of the currently known six members of the IL-17 cytokine family and is implicated in immune responses to infectious pathogens and in the pathogenesis of inflammatory autoimmune diseases like psoriasis. Psoriatic skin is characterized by high expression of IL-17A and IL-17F, which act on immune and non-immune cell types and strongly contribute to tissue inflammation. In psoriatic lesions, IL-17A, IL-17E, and IL-17F are involved in neutrophil accumulation, followed by the formation of epidermal micro abscesses. IL-17A together with other Th17 cytokines also upregulates the production of several chemokines that are implicated in psoriasis pathogenesis. IL17A-targeting antibodies show an impressive clinical efficacy in patients with psoriasis. Studies have reported an improvement of at least 75% as measured by the psoriasis area and severity index (PASI) in >80% of patients treated with anti-IL-17A therapy. Psoriasis skin manifestations, cardiovascular as well as metabolic disease in psoriasis appear to share pathogenic mechanisms evolving around IL-17A and its proinflammatory role. Thus, anti-IL-17A therapy not only improves skin manifestations of psoriasis, but also cardiovascular inflammation as well as metabolic factors and different domains of psoriatic arthritis (PsA) including peripheral arthritis, enthesitis, dactylitis, and axial involvement. This review summarizes the biological role of IL-17A, before reviewing currently available data on its role in the physiology and pathophysiology of the skin, as well as the cardiovascular and the metabolic system. In conclusion, clinical recommendations for patients with moderate to severe psoriasis based on the current available data are given.

## Introduction

Increased cardiovascular mortality is a common feature amongst many chronic inflammatory disorders. Interestingly, several lines of evidence suggest that effective systemic anti-inflammatory therapy may reduce this risk (1–6): A recent large randomized study showed that canakinumab, an antibody blocking interleukin (IL)-1β, is protective against myocardial infarction in a population at risk whereas its immunosuppressive effect also exposed patients to an increased risk of infections (7). Also, it is a well-known clinical observation that inflammatory diseases are accompanied by metabolic implications such as hyperglycemia, insulin resistance, and increased fatty acids (8). On the other hand, several metabolic diseases have inflammatory implications. Most important, there is a chronic and low-grade state of inflammation (“metaflammation”) in obesity and type 2 diabetes, both systemically and locally in visceral adipose tissue (“adipoflammation”) and other tissues (9–11). Antagonistic therapeutic approaches to proinflammatory molecules such as IL-1β, IL-1 receptor (IL-1R), tumor necrosis factor (TNF), and C-C chemokine ligand (CCL)2 were shown to improve glycemic control and metabolic parameters in patients with type 2 diabetes (12).

Psoriasis—one of the most common skin diseases with a prevalence of ~2%—is amongst the few non-communicable diseases the World Health Organization identified as a major global health problem (13). This is due to its chronic course, stigmatizing character due to the readily visible red scaly plaques, and its association with numerous other major diseases, such as hypertension, myocardial infarction, or diabetes (14). Over the last decade, targeted therapies have substantially improved our capacity to reduce signs and symptoms of psoriasis patients to an extent that—with the advent of biologics blocking IL-17A—“clear skin” became a feasible treatment goal (15). As strategies to block interleukin 1 have not been effective in treating psoriasis (16), the question arises whether IL-17A or other cytokines like TNFα, IL-12/23 (p40), or IL-23 might be a therapeutic target that would allow achieving therapeutic effects beyond the skin and joints, namely regarding the cardiometabolic situation of the patients.

In this review, we will briefly summarize what is known about the biological role of IL-17A, before reviewing currently available data on its role in the physiology and pathophysiology of the skin, the cardiovascular, and the metabolic system. Finally, we will share our point of view regarding the research agenda in the field and possible therapeutic implications.

## Biology of IL-17A and IL-17F

The IL-17 cytokine family is formed by six members. As numerous other cytokines, IL-17 cytokines are part of the adaptive and innate immunity (17). IL-17A and IL-17F are significantly implicated in immune responses to infectious pathogens and in the pathogenesis of inflammatory autoimmune diseases like psoriasis. Furthermore, evidence indicates an additional proinflammatory activity of IL-17C and IL-17E within the pathogenesis of psoriasis (18–21).

IL-17A and IL-17F share the highest structural homology inside the IL-17 cytokine family and by this fulfill very similar biological functions (22). IL-17A/IL17F homo- and heterodimers bind to heterodimeric receptors (IL-17R) (23). The IL-17RA unit is part of the receptors for IL-17A, IL-17F, and IL-17E. Downstream of the IL-17R, signaling proteins are activated that result in the activation of transcription factors like nuclear factor kappa B (NFκB), activator protein 1 and CCAAT-enhancer-binding protein. One protein that transmits the signals from the IL-17R at an early stage is the NFκB activator 1 (ACT1) (24). Of note, the gene encoding ACT1 TNF receptor-associated factor 3 interacting protein 2 (TRAF3IP2) is one of the genes with common variants that are highly associated with the susceptibility to psoriasis and psoriatic arthritis (24).

Several factors have been reported to induce IL-17A and IL-17F expression in lymphoid cells. This has been intensively studied in CD4^+^ T helper (Th) cells. The critical cytokines that promote the differentiation of IL-17-producing Th (Th17) cells from naive Th cells are typically combinations of TGF-β1 with STAT3-activating cytokines like IL-6 or IL-21 (25). Cytokines like IL-1β and IL-23 can potentiate IL-17A and IL-17F expression. Moreover, the combination of IL-6, IL-1β, and IL-23 can induce IL-17A and IL-17F production in Th cells (25). The latter cytokine, IL-23, is responsible for the pathogenicity of autoreactive Th17 cells and in addition promotes IL-17 production in different lymphoid cell types including CD3^+^CD4^−^CD8^−^ populations as well as innate lymphoid cells (26, 27).

## IL-17A in Dermatology

### Organ-Specific Pathophysiology

IL-17A-producing T cells can be protective for the organism but depending on their target cells can also cause tissue damage. Such diverse effects of IL-17A become evident in skin immunity. Effective immune responses against cutaneous pathogens require IL-17A expression. Examples are skin infections with bacteria like *Mycobacteria tuberculosis* or with fungi like *Candida albicans* (28, 29). IL-17A contributes to pathogen defense mechanisms by promoting neutrophil recruitment or production of antimicrobial peptides. However, high levels of IL-17A released during autoinflammatory or autoimmune conditions can also result in pathological skin changes. For instance, IL-17A has been reported to be present in *Hidradenitis suppurativa*, a devastating neutrophil-rich inflammation with formation of nodules and abscesses (30). The best studied inflammatory skin disease with a predominant role of IL-17A is psoriasis.

As confirmed in many studies, psoriatic skin is characterized by high expression of IL-17A and IL-17F (31). More recently, other IL-17 family members, namely IL-17C and IL-17E, have been reported to be expressed in psoriatic skin (18). While IL-17A and IL-17F are mainly produced by immune cells (e.g., Th cells, γδ T cells), the cellular sources of IL-17E also include epithelial cells like keratinocytes. Other sources comprise neutrophils and mast cells (32).

IL-17A and IL-17F act on immune and non-immune cell types and strongly contribute to tissue inflammation. For instance, they are involved in neutrophil recruitment. In the skin of psoriatic lesions, neutrophil accumulation is followed by the formation of epidermal micro abscesses (33, 34). IL-17 together with other Th17 cytokines also upregulates the production of several chemokines that are implicated in psoriasis pathogenesis, including CCL20, C-X-C motif chemokine ligand (CXCL)8, or CXCL1 (33, 34). Likewise, the production of matrix metalloproteinases (MMP) like MMP9 and antimicrobial peptides like β-defensin 2, LL37, and S100 proteins are controlled by IL-17A and IL-17F.

### Experimental and Clinical Evidence for a Role of IL-17A in Psoriatic Inflammation

First antibodies that target IL-17A or its receptor IL-17RA are approved for the treatment of psoriasis. Secukinumab and ixekizumab both neutralize IL-17A, while brodalumab binds to the IL-17RA. The clinical efficacy of these IL17-targeting antibodies in psoriasis is demonstrated in several randomized studies and registries, as a clinical improvement of at least 75% as measured by the PASI is observed in >80% of patients treated (35–37). Similar PASI responses are achieved in clinical trials testing antibodies neutralizing IL-23 (p19), which is a potent inducer of IL-17A production by immune cells (38–40). In the recent five years there has been growing clinical evidence that IL-17A inhibitors are effective and safe in treating moderate to severe plaque type psoriasis.

A fully human monoclonal antibody that selectively neutralizes IL-17A, a key cornerstone cytokine involved in the development of psoriasis, is an approved treatment for patients with moderate to severe plaque psoriasis. Almost 10 years ago, the first clinical trial data with secukinumab were published, and at an early stage it was obvious that the targeted treatment with IL-17A antagonists has a higher capability of improving psoriasis compared to established previous therapy options. The ultimate treatment goal of PASI 75 (75% decrease of symptoms compared to baseline) and improvement of quality of life as assessed by dermatology life quality index (DLQI) of <5 was easily achieved by almost all patients treated with secukinumab (35). Furthermore, the IL-17A inhibitor secukinumab 300 mg s.c. treatment in head-to-head double-blind randomized trials was superior to established biological treatments like etanercept (TNF inhibitor) and ustekinumab (anti-IL12/23), raising the bar, where more than 80% of the patients achieved a PASI 90 and DLQI of 0/1 (35, 41, 42).

Beside short-term data, secukinumab has shown long-lasting efficacy and safety. Recently, high sustained efficacy and safety of 300 mg secukinumab treatment through 5 years in moderate to severe psoriasis was published (43). Direct comparison with first-line conventional treatment in Germany with fumaric acid esters showed clinically meaningful and statistically highly significant differences in patients naïve to any previous systemic treatment (44). Early treatment with secukinumab even as a first-line systemic treatment became possible, not only according to label but also in daily practice. The recent German guideline recommends secukinumab in patients with moderate to severe psoriasis, who in the judgment of the dermatologist have less chance to respond to other first-line conventional systemic therapies (45); other agents blocking the IL-17 pathway had not been approved for use at the time of guideline publication. Secukinumab has shown efficacy and safety in the complete spectrum of psoriasis manifestations, including nails, scalp, palms and soles, joints and symptoms like itch, scaling, and skin redness (46, 47).

Ixekizumab is a humanized monoclonal antibody with a selective inhibition of IL-17A and approved for the treatment of moderate to severe psoriasis. After initial s.c. treatment with 160 mg, followed by 80 mg every other week in the first 12 weeks, ixekizumab showed similar high PASI 90 responses as other IL-17 inhibitors in direct comparison to etanercept and ustekinumab (48, 49). Also, long-term 4-year continuous treatment with ixekizumab has recently been presented (50). Ixekizumab has also received first line approval from the EMA, but due to its late approval date has not yet been included in the most recent guidelines.

Clinical studies with anti-IL-17 (ixekizumab and secukinumab) are ongoing in adolescents and children, and results are not yet presented.

Brodalumab, a monoclonal antibody with high binding affinity to IL-17 receptor A (IL-17RA), is a treatment modality approved by the FDA for second line and by the EMA for the first line treatment of moderate to severe psoriasis. Treatment with 210 mg every 2 weeks shows very rapid onset of response with high clearing capacity where ~40–50% of treated patients had a PASI 100 (51). Up to now, there is no approval for PsA.

Importantly, targeting IL-17A is also effective in psoriatic arthritis, demonstrating that the psoriatic inflammation at distant sites also depends on the action of IL-17A (52). Also, different domains of PsA showed response to secukinumab including peripheral arthritis, enthesitis, dactylitis, and axial involvement (53, 54). In line, ixekizumab also improved different domains of psoriasis and PsA (55).

All IL-17 inhibitors have a similar safety profile, which is comparable with other approved biologics. Unlike TNF-alpha inhibitors, an increased risk of tuberculosis occurrence or reactivation was not observed. Unique for this biologic group is an increased risk for muco-cutaneous candida infection, mostly mild or moderate and easily manageable (56). Patients with active chronic inflammatory bowel disease (IBD) should not receive IL-17 inhibitors because of potential aggravation of IBD. Nevertheless, latest analyses of 21 clinical trials showed incidence rates of IBD comparable to untreated patients with psoriasis or psoriatic arthritis (57). Newest real-life data are confirming safety and tolerability of the IL-17 inhibitors also in daily practice, mirroring the evidence from clinical trials (58).

Currently, the effects of IL-17A neutralization on psoriasis-associated comorbidities are being investigated. Psoriasis patients with metabolic syndrome seem to have higher serum levels of IL-17, leptin, and c-reactive protein (CRP), while adiponectin levels seem to be lower (59–61). One could assume that the systemic suppression of IL-17 levels may be of benefit for patients with comorbidities like metabolic syndrome and cardiovascular disease.

### Potential Therapeutic Implications

A common feature of IL-17 inhibitors is their fast mode of onset. Therefore, they might be particularly well-suited to treat patients with wide-spread and/or active/non-stable disease. Looking at the two currently available antibodies directly targeting IL-17A—secukinumab and ixekizumab—these still seem to be sufficiently different from each other to validate an attempt to use one in case the other has failed. There is an increasing number of reports of patients who did respond to ixekizumab after having failed to respond to secukinumab (62).

The third molecule currently available with a related mechanism of action, brodalumab, targets the IL-17RA chain. This results in inhibition of effects mediated by IL-17A as well as additional members of the IL-17 family. This mode of action might have two clinically relevant consequences:

1. As other IL-17s also play a pro-inflammatory role in psoriasis (17, 18), the efficacy of brodalumab might be particularly high. Indeed, indirect comparisons point into this direction (63).
2. Blocking the IL-17 receptor might result in a feedback-loop with substantial or even higher amounts of the mediator present. Therefore, drug withdrawal might result in a fast relapse of the disease. This has indeed been observed (64). Consequently, brodalumab may need to be given continuously, or switched directly to another active treatment.

## Cardiovascular Inflammation in Psoriasis

### Experimental and Clinical Evidence

An association of increased cardiovascular disease with psoriasis has been known for a long time, and the incidence of thromboembolic events in patients with untreated psoriasis has long been known to be higher than in age-matched controls (65). In addition, severe psoriasis is associated with a 50% increased risk of cardiovascular mortality (66), and life expectancy in patients with severe psoriasis is reduced (females: −4.4 years, males: −3.5 years) (67). Complicating the identification of a cause-effect relationship, it is also known that psoriasis patients are more likely to have an increased body mass index (68), are more likely to have metabolic syndrome (69) and type 2 diabetes (70) as well as depression (71, 72). Thus, one cannot exclude that elevated cumulative cardiovascular risk factors contribute to their increased rate of cardiovascular disease, even though evidence from large studies supports the existence of an independent relationship between psoriasis and cardiovascular disease (73, 74).

Recent evidence indicated that IL-17A may represent one of the main links between cardiovascular disease manifestations and psoriatic inflammation. Mouse models for psoriasis demonstrate that psoriasis is a T cell-mediated disease in which—among other factors—IL-17A is released from skin-infiltrating T cells to induce pathology via neutrophil recruitment (75–77). A T cell-mediated production of IL-17A has also been shown for human psoriasis (78).To mimic this situation and to study downstream events, mice overexpressing IL-17A only in keratinocytes have been generated (79). On their back skin, these mice developed skin lesions with extensive scaling and epidermal barrier loss. Histologically, the skin showed typical features of human psoriasis with hyperkeratosis, acanthosis, and infiltration with T cells and neutrophils. CD4^+^ T cells infiltrating the skin carrying a Th17 signature were abundant. Interestingly, the life span of these K14-IL-17^ind^ mice was significantly shorter, and premature death started to occur at around 4 weeks of life, with most of the mice having died after 6 months (80). A thorough investigation established that hypertension, resulting in an increased heart/body ratio with cardiomyocyte hypertrophy, and a pathologic endothelial function were typical features of this murine model compatible with an increased cardiovascular risk. Signs for other cardiovascular risk factors such as increased body weights, hyperlipidemia, and/or hyperglycemia were not found.

To establish a direct link between the psoriatic phenotype and cardiovascular death, treatment with anti-TNF-α or anti-IL-6 was performed (80). Interestingly, these anti-inflammatory treatments not only efficiently reduced skin inflammation, they also abrogated pathologic endothelial stiffness, suggesting that the systemic inflammation present in K14-IL-17A^ind^ mice was directly responsible for cardiovascular death. These results also suggested that IL-6 and TNF-α act downstream of IL-17A to promote cardiovascular comorbidity (80). In line with this, in recent reviews, a strong link between vascular inflammation, neutrophil recruitment, and IL-17 in mouse models has been highlighted (81, 82).

The CARIMA (Evaluation of Cardiovascular Risk Markers in Psoriasis Patients Treated with Secukinumab) study was a 52-week, randomized, double-blind, placebo-controlled, exploratory trial in patients with moderate to severe plaque psoriasis (83). Patients with prior cardiovascular disease were excluded. The primary outcome was endothelial function measured by flow-mediated dilation (FMD). In line with prior observations (84, 85), baseline FMD in the 151 psoriasis patients included was significantly lower in psoriasis patients than healthy volunteers, suggesting that subclinical alterations of endothelial function are present even in the absence of measurable clinical manifestations. At week 12, baseline adjusted mean FMD was higher in patients receiving secukinumab vs. those receiving placebo, but this difference did not reach significance. At week 52, the FMD was significantly higher than at baseline in patients receiving secukinumab for 52 weeks (+2.1%). Other relevant CV markers were unchanged. Thus, CARIMA indicates that secukinumab might have a beneficial effect on CV risk by improving the endothelial function of patients with plaque psoriasis. In line, adalimumab induced improvement of FMD in 14 psoriasis patients (84). Other studies with small patient cohorts showed mixed or no effect on FMD after anti-TNF treatment (85, 86). All in all, the increase in FMD at week 52 in anti-IL-17A-treated patients in the CARIMA study may be clinically relevant, since a 1% increase in absolute FMD is associated with a 13% decrease in the relative CV risk (87).

Other studies assessed the carotid plaque burden in psoriasis patients during biologic treatment (88, 89). Interestingly, psoriasis patients receiving anti-TNF treatment exhibited decreased vascular inflammation and a gender-dependent effect on carotid plaque progression after 1 year (88). Further evidence of the effect of anti-IL-17A treatment on cardiovascular disease in psoriasis was provided by Elnabawi et al. (89). In this prospective observational study, 121 patients without prior systemic therapy received treatment with various biologicals for 1 year; 169 patients with topical and/or phototherapy served as controls. Before and after treatment, total coronary plaque burden and subcomponents in coronary computed tomography angiography were determined. Interestingly, biologic therapy was associated with a 5% reduction of non-calcified plaques, a reduction of the necrotic cores, and no effect on fibrous burden. Compared to anti-TNF or anti-IL-12/23 treatment, anti-IL-17A was most efficient in reducing non-calcified plaque burden (12% compared to 5 and 2%, respectively).

### Potential Therapeutic Implications

The effect of biologic treatment on subclinical cardiovascular disease in psoriasis patients is unclear so far. Some studies have convincingly shown improvement after therapy and others showed no changes; the majority of studies have assessed the effect of anti-TNF treatment in small patient collectives. However, the most recent studies (83, 89) with highly sensitive parameters for cardiovascular surrogate parameters, and comparably large patient cohorts as well as control groups have indicated that anti-IL-17A treatment (and potentially other biologics) may represent a promising agent to alter not only skin disease, but also cardiovascular comorbidity. Larger, randomized trials addressing this effect are needed.

## IL-17A in the Cardiovascular System

The improvement of cardiovascular disease might be a direct consequence of a specific or relatively unspecific anti-inflammatory therapy in general. A first prospective, randomized, placebo-controlled, phase-III study could demonstrate in high risk patients after myocardial infarction that specific neutralization of IL-1 by canakinumab (CANTOS trial) can significantly reduce cardiovascular mortality (7). In contrast, a relatively unspecific anti-inflammatory therapy by methotrexate (CIRT trial) failed to achieve significant effects on cardiovascular mortality (90). Based on these considerations, highly specific and molecular therapies targeted against single cytokines or their receptors such as the IL-17A system appear to be promising regarding cardiometabolic comorbidities in psoriasis.

### The Role of Inflammatory Cytokines in the Pathogenesis of Atherosclerosis

Atherosclerosis and thromboembolic diseases are maladaptive chronic inflammatory diseases whose pathogenesis and progression are determined by an interaction between immuno-inflammatory mechanisms, hemodynamic disturbances, activation of platelets and humoral coagulation factors, and lipid accumulation. The resulting ischemic diseases (e.g., myocardial infarction, cardiomyopathies or stroke) represent a prognosis-relevant comorbidity frequently associated with most inflammatory conditions, including those, like psoriasis, in which IL-17A appears to play an important role (91–93).

Interestingly, the pathophysiology of atherosclerosis and psoriasis share common aspects (94, 95). These similarities include an inflammatory background associated with local formation of modified autoantigens that are targeted by both the innate and adaptive immune system and in which Th1 cells have an important initiator function. The extravasation and migration into the vessel wall of these inflammatory cells is initially stimulated by adhesion molecules whose production is controlled by the secretion of pro-inflammatory cytokines and chemokines in response to alterations in the laminarity of blood flow. Several cytokines such as IL-2, interferon gamma (IFN-γ), and, most importantly, TNF-α, have important modulatory functions. Of note, the same mediators are also known to be linked to endothelial dysfunction and vascular calcification in other inflammatory diseases such as autoimmune diseases, inflammatory bowel syndromes, and rheumatoid arthritis. Cytokines and mediators including TNF-α, IL-1β, IL-6, and IFN-γ, along with MMP, are also responsible, upon stimulation, of the destabilization of atherosclerotic plaques, leading to ischemic events (96).

### Experimental and Clinical Evidence for Proatherogenic and Atheroprotective Effects of IL-17A

**Figure 1:**
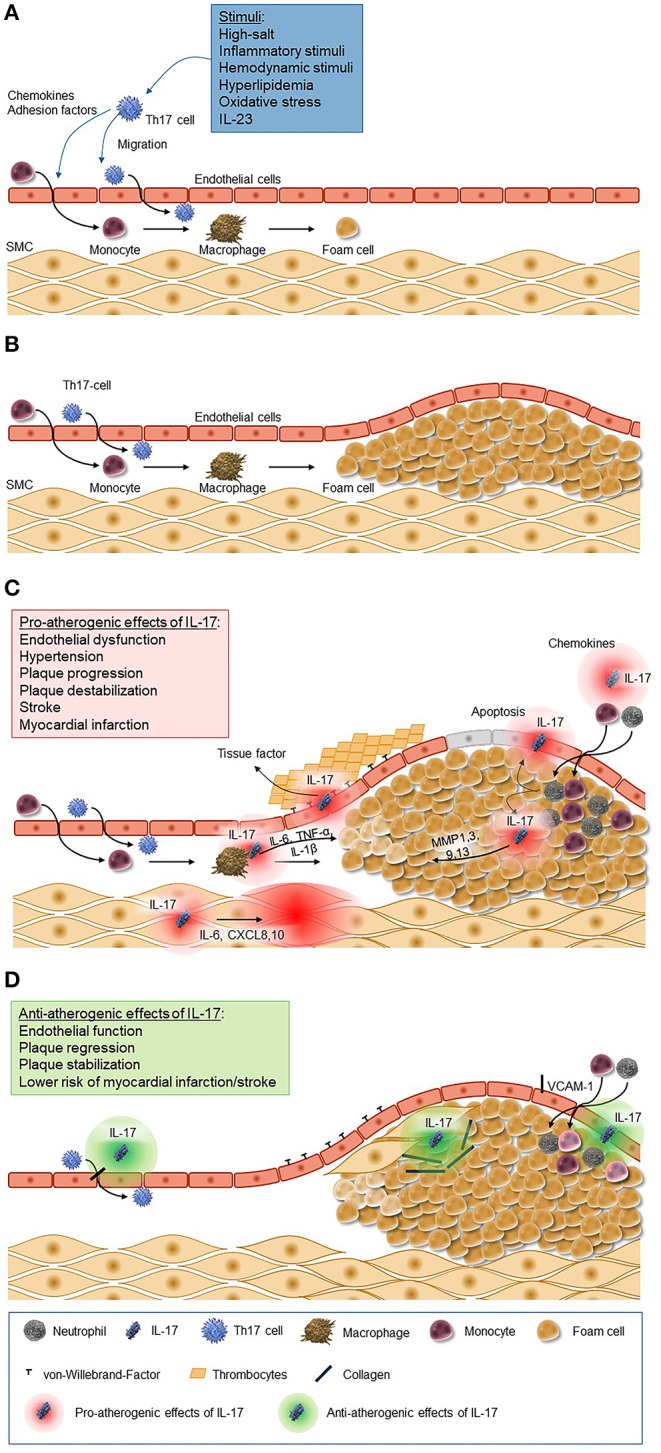
Pro-atherogenic and anti-atherogenic effects of IL-17. **(A)** Chemokines and adhesion factors are secreted by Th17 cells and cells of the innate immune system in response to a variety of stimuli (e.g., inflammatory stimuli, hemodynamic stimuli, and hyperlipidemia). These chemokines trigger migration and differentiation of monocytes and Th17 cells through the epithelium, which leads to the activation of macrophages and macrophage-mediated foam cell formation. **(B)** Foam cells accumulate to plaques as a key process of atherosclerosis. **(C)** IL-17 induces the release of chemokines leading to the recruitment of neutrophils and monocytes to the atherosclerotic lesion and their transformation to macrophages and foam cells and accumulation in fatty streaks. Further it stimulates macrophages to produce inflammatory cytokines such as IL-6, TNF-α, and IL-1β, matrix metalloproteinase from fibroblasts, endothelial cells, and epithelial cells, and it induces apoptosis of vascular endothelial cells. The increased secretion of endothelium-mediated von-Willebrand-factor promotes platelets adhesion and aggregation. **(D)** IL-17, however, also has antiatherogenic effects as it stimulates the proliferation of smooth muscle cells and the production of collagen and inhibits the expression of adhesion molecules. CXCL, C-X-C motif chemokine ligand; IL, interleukin; MMP, matrix metallopeptidase; SMC, smooth muscle cells; VCAM, vascular cell adhesion molecule.

The concept that IL-17 might have a pro-atherogenic role is supported by several lines of evidence (Figures 1A–C). The initial activation of Th17 cells may result from a variety of mechanisms, including inflammatory stimuli triggered by lipid accumulation, external inflammatory stimuli, or hemodynamic conditions. Additionally, exogenous stimuli, like sodium salt load, might also stimulate the induction of Th17 cells as shown recently in murine and human assays (97). Importantly, the IL-17A receptor is ubiquitously expressed in the vessel wall; among the pleiotropic activities of this receptor the induction of the production of TNF-α, IL-1β, CCL2, and adhesion molecules like intercellular adhesion molecule 1 (ICAM-1) provide important links to the pathogenesis of atherosclerosis (98). In a hypercholesterolemic rat model, IL-17A inhibition was associated with a 50% reduction of the atheroma area and with a reduction in the maximum percent stenosis, and it lead to lower expression of the chemokine CCL5 and lower intraplaque levels of IL-6, TNF-α, adhesion molecules such as vascular cell adhesion molecule 1 (VCAM-1), and the prothrombic molecule TF; in addition, it was associated with features suggestive of reduced plaque vulnerability such as an increase in intravascular smooth muscle cells and in the collagen content of the fibrous cap, a reduced expression of metalloproteinases, and a reduced apoptosis in the lesion. Further, cytokine/chemokine expression was reduced, suggesting a modulatory role of IL-17A in vascular inflammation (99). These data were confirmed in similar murine models in which exogenous application of recombinant IL-17A stimulated pathological changes associated with increased plaque instability, and, vice versa, IL-17A inhibition produced a regression of atherosclerosis (100–103).

Several researchers have shown that IL-17A is also required for the development of ischemic and dilated cardiomyopathy (DCM), since Th17 cells have been observed to infiltrate the heart during experimental autoimmune myocarditis (104, 105). IL-17RA^−/−^ mice were protected from inducing DCM, although they developed myocarditis comparable to WT controls (104). IL-17RA^−/−^ mice had diminished infiltration of neutrophils, which also have been shown to play an important role in the recruitment of monocytes (106, 107). In this model, cardiac fibroblasts were stimulated by IL-17A to produce key cytokines and chemokines that are critical downstream effectors in the recruitment and differentiation of myeloid cells. The authors concluded that IL-17A, while less important for the early stages of myocardtis, is required for the progression to DCM (105). These results were similar to the findings of Liu et al. who showed that IL-17 contributes to cardiac fibrosis and suggested that myocardial fibrosis induced by IL-17 is dependent on the PKCß/ERK1/2/NF-κB signaling pathways (108). In addition, Yuan and colleagues found increased numbers of Th17 cells in the blood of patients with DCM and an elevated level of Th17 cytokines in their serum (109).

Other research demonstrated that IL-17A mediates inflammation and the inappropriate increased vascular superoxide (${\text{O}}_{2}^{-}$) production in response to high-fat diet-induced atherosclerosis, one of the crucial determinants of endothelial dysfunction in the setting of vascular disease (102). *Ex vivo*, IL-17A stimulation of human plaque fragments induced a pro-inflammatory, pro-thrombotic, plaque destabilizing and cell-attracting phenotype (110); *in vivo* in humans, higher IL-17A plasma levels were found in patients with coronary artery and carotid disease (111), an observation which might however be interpreted both as causation or as activation of compensatory mechanisms. Importantly, in these studies, the expression of IL-17A was maximal in human carotid artery plaques derived from symptomatic patients with stroke or transient ischemic attack, a piece of evidence that fits well with the concept that IL-17A is associated with plaque instability (112). Even further, a study in coronary thrombus aspirates from patients who had suffered an acute myocardial infarction demonstrated that up to 10–30% of the occluding thrombus mass is represented by neutrophil extracellular traps; in this study, IL-17A and IL-17F appeared to be important constituents of fresh, but not chronic, thrombi, indicating a possible role also for IL-17-dependent inflammation in acute thrombosis (113).

From a functional standpoint, IL-17A was also shown to promote endothelial dysfunction and angiotensin II-induced hypertension in a recent publication: in this murine model, angiotensin II infusion increased IL-17 production from T cells, and IL-17 knockout mice did not develop sustained hypertension, endothelial dysfunction, or evidence of vascular oxidative stress after chronic infusion of this potent vasoconstrictor (114). In another mouse model of IL-17A and enhanced green fluorescent protein (EGFP) co-overexpression in keratinocytes simulating clinical psoriasis, evidence of vascular dysfunction (including increased cardiovascular mortality) and arterial hypertension, along with large aortic wall cellular infiltrates, was observed. IL-17 may also contribute to atherogenesis by inducing the maturation and differentiation of macrophages; the subsequent activation of these precursors of foam cells by oxidized low-density lipoprotein (oxLDL) is considered to be the first step in the formation of atherosclerotic plaques (115).

Despite this convincing evidence, however, some uncertainty remains, as other publications have shown significantly smaller atherosclerotic lesions in mice with increased IL-17 expression (an observation that was reversed with IL-17 inhibition) and an association between IL-17 expression and plaque stability in human carotid artery plaques (116). Although some of the differences observed across different studies can be at least partially explained by differences in the study design, the method used to inhibit IL-17A, the animal model, and the site of the atherosclerotic lesion, the existence of controversial results needs to be acknowledged. Finally, evidence that IL-17A might also have atheroprotective effects also exists (Figure 1D): low serum levels of IL-17A have been associated with a higher risk of cardiovascular recurrences in coronary artery disease patients, an observation which might suggest that IL-17A might exert some form of preconditioning-like protection, and a murine IL-17A knockout model conferred resistance to high-fat diet-induced weight gain (102). Similar results were also obtained by Simon et al. using data from 981 patients with acute myocardial infarction, demonstrating that low serum levels of IL-17 and high soluble VCAM-1 levels are associated with a higher risk of major cardiovascular events of death and recurrent myocardial infarction. They concluded that these results raise possible concern about the use of inhibitors of the IL-17 pathway in clinical settings associated with a high cardiovascular risk (117).

Finally, a large meta-analysis including 38 randomized controlled trials showed no significant difference in risk of major adverse cardiovascular events (myocardial infarction, cerebrovascular accident, or cardiovascular death) in patients with psoriasis (*n* = 18,024) treated with biologic therapies including anti-IL-12/23, TNF-α inhibitors, and anti-IL-17A agents (118).

### Potential Therapeutic Implications of Anti-inflammatory Therapy on Cardiovascular Function

From a therapeutic perspective, preliminary data show that pharmacological inhibition of cytokines is associated with a significant improvement in endothelial function: for instance, a single subcutaneous injection of the TNF-α inhibitor etanercept is associated with an improvement in flow-mediated dilation in postmenopausal women (119), and a preliminary study reported a significant improvement in resting endothelial function after therapy of psoriasis with anti-TNF-α and anti-IL-12p40, suggesting an activation of resting endothelial function in response to anti-inflammatory therapy (Karbach, unpublished). Whether this also occurs with a specific inhibition of IL-17A was tested in the CARIMA study described above, which showed a significant improvement after 54, but not 12, weeks of therapy with secukinumab (83). In sum, psoriasis and vascular disease appear to (at least partially) share common pathogenetic mechanisms, and an anti-inflammatory therapy (with a focus on the IL-17A signaling cascade) appears to not only reduce the skin manifestations, but also to improve (cardio)vascular function.

## IL-17A in the Metabolic System

Besides LDL cholesterol (*lipocentric view*), the main metabolic factors causing cardiovascular complications (discussed in chapter 5) comprise hyperglycemia (*glucocentric view*) and hyperinsulinemia (insulin resistance, either as overt type 2 diabetes or as pre-diabetes). These two conditions become strongly activated during inflammation (and also during infection) via the so called “energy appeal reaction.” The molecular basis of this energy appeal reaction has been evolutionarily conserved and is characterized by an immuno-metabolic crosstalk leading to insulin resistance. Since the adipose tissue as a central organ in insulin resistance as well as adipokines, chemokines and cytokines such as IL-17A are involved in these mechanisms, the following chapter will summarize the underlying molecular mechanisms.

### Effect of Inflammatory Processes on Insulin Resistance

During 600 million years of evolution, *Drosophila*'s fat body developed into distinct organs such as adipose tissue, liver, and cells of the hematopoietic and the immune system (120). Since all of these organs were initially combined in this fat body, there must have been a molecular crosstalk between the metabolism on one hand and the immune system on the other hand. This kind of interaction has been conserved and is still present in humans (121). The molecular cross-talk between both systems represents a central homeostatic mechanism that is regulated by common pro-inflammatory molecules with a dichotomous function such as TNF, IL-6, IL-1, and many others (10, 122, 123). As demonstrated and reviewed previously (121), there is an additional need of up to 500 kJ for activation of the monocytic cell pool during inflammation. Energy has to be re-allocated to the cells of the immune system (energy appeal reaction) and this re-distribution is mediated by induction of systemic and local insulin resistance of tissues such as liver, muscle, and fat (124). Taken together, physiological insulin resistance during infection or inflammation re-distributes glucose and fatty acids to cells of the immune system. This situation perfectly resembles type 2 diabetes. Reduced insulin sensitivity as well as reduced insulin secretion are culprits in type 2 diabetes. Levels of glucose and fatty acids are chronically elevated. Glucotoxicity and lipotoxicity are the main mediators of diabetic complications (125).

Most importantly, there is a high grade of dynamic plasticity (126) of visceral adipose tissue. In the obese state, a shift from anti-inflammatory M2 macrophages (responsible to IL-4, IL-13; secreting IL-10) to proinflammatory M1 macrophages (responsible to lipopolysaccharide, IFN-γ; secreting IL-6 and TNF) occurs. Generally, local CD4^+^ T cells are being replaced by CD8^+^ T cells, and increasing numbers of granulocytes and even eosinophils can be registered (127–130). Moreover, specific CD4^+^ T cell subsets such as IL-17-secreting Th17 cells and IL-22-producing Th22 are infiltrating the adipose tissue and represent local mediators of inflammation and insulin resistance (127–130). There is also a shift from anti-inflammatory adipokines such as adiponectin to proinflammatory adipokines such as leptin or resistin (12, 126). Taken together, adipoflammation mediates local and systemic insulin resistance as well as metabolic inflammation.

There are several common immuno-metabolic signaling pathways in adipocytes, macrophages, and pancreatic beta-cells such as the Toll-like receptor-(TLR-)2, TLR-4, NFκB pathway, and the inflammasome/caspase-1/IL-1β pathway (12). Macrophage-derived IL-6 for example upregulates lipolysis in adipocytes leading to an increased flux of fatty acids and glycerol into the liver (12). These mechanisms upregulate hepatic glucose production, a main finding in diabetes. It is important to remember that insulin receptor signaling does not work well when cytokines and lipids are around. Lipids and cytokines are known to activate certain types of kinases that cause an inhibitory phosphorylation of insulin receptor substrate-(IRS-)1 and IRS-2 (120). Phosphorylation takes place at the wrong sites leading to less effective insulin signaling. Taken together, the most important finding of metabolic research during the last decades is given by the fact that nutrients are able to act through pathogen-sensing pathways. Similarly, stress pathways such as hypoxia, reactive oxygen species (ROS), endoplasmic reticulum activation, lipids, fatty acids, and cytokines are integrated with insulin signaling via inhibitory mechanisms by c-Jun N-terminal kinase (JNK) activation (120). JNK represents the central kinase connecting stress and inflammation with insulin signaling. As a conclusion, inflammation, and stress regularly induce insulin resistance by common mechanisms.

Leptin and IL-17 represent a suitable molecular example for the reciprocal regulation between immune system and energy metabolism. Mice with a T cell-specific deficiency of hepatocyte progenitor kinase-like/germinal center kinase-like kinase (HGK, also termed MAP4K4) develop systemic inflammation together with insulin resistance, a condition that could be ameliorated by neutralization of IL-6 or IL-17 (130). In this model, it was proven that HGK downregulates IL-6 production in T cells via phosphorylation and subsequent degradation of TNF-receptor-associated factor-2 (TRAF2) (130). Moreover, HGK-deficient and IL-6-overproducing T cells undergo differentiation into IL-6/IL-17-co-expressing cells that accumulate in adipose tissue (130). Accumulation of these cells in adipose tissue enhances the release of leptin from adipocytes, which in turn cooperates with IL-6 to induce Th17 differentiation. Local and systemic insulin resistance are driven by this coregulation.

### IL-17 Links Inflammation With Insulin Resistance and Adipocyte Dysfunction

From an epidemiological point of view (69, 131–133), obesity and psoriasis are connected via the HLA-Cw6 locus (134), and obesity is ~35-fold more common in psoriasis. Vice versa, in patients with severe psoriasis, obesity and metabolic syndrome are more common (1.79-fold and 2.26-fold, respectively). Prevalence of the metabolic syndrome increases with increasing severity of psoriasis (135). With respect to diabetes (133, 136, 137), higher rates of psoriasis (~1.76-fold), especially of severe psoriasis (~2.1-fold), have been reported, and elevated levels of TNF seem mainly to be responsible via JNK activation and subsequent IRS-1 phosphorylation (138).

**Figure 2:**
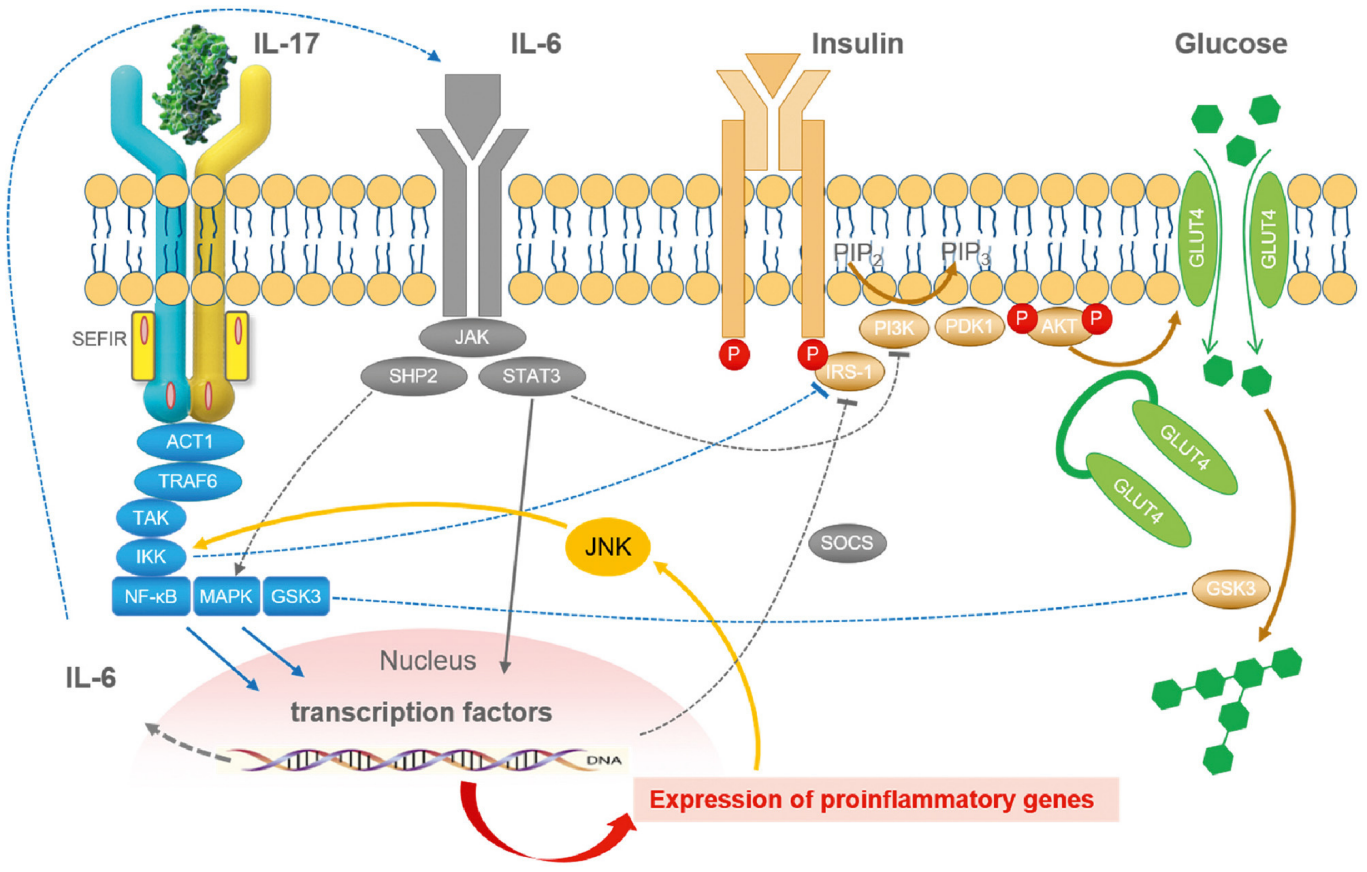
The integrated role of IL-17 in the cytokine- and inflammation-related regulatory network of insulin signaling. In general, inflammation reduces insulin sensitivity and insulin receptor signaling via IRS-1 by a myriad of different mediators. Cellular glucose disposal needs glucose transport via specific glucose transporters such as GLUT4 in adipose tissue and in striated muscle. Activation of the NFκB and the JNK pathway via different lipids and cytokines such as IL-6 and IL-17 (but also TNF, IL-1, and others) decrease insulin signaling. Lipids and cytokines are known to activate certain types of kinases that cause an inhibitory phosphorylation of IRS-1. Phosphorylation takes place at the wrong sites leading to less effective insulin signaling. Stress pathways such as hypoxia, ROS, ER activation, lipids, fatty acids, and cytokines are all integrated with insulin signaling via inhibitory mechanisms by JNK activation. Taken together, inflammation and stress always induce insulin resistance by common mechanisms. JNK represents the central kinase connecting stress and inflammation with insulin signaling. ACT1, nuclear factor-kappa-B activator; AKT, protein kinase AKT; ER, endoplasmic reticulum; Glut4, glucose transporter-4; GSK3, glycogen synthase kinase-3; IKK, I-kappa-B-kinase; IRS, insulin receptor substrate; JNK, Janus kinase; MAPK, mitogen-activated protein kinase; PDK1, pyruvate dehydrogenase kinase-1; NFκB, nuclear factor-kappa-B; PI3K, phosphoinositol-3-kinase; ROS, reactive oxygen species; SEFIR, similar expression to FGFR1; SHP2, tyrosine phosphatase SHP2; SOCS, suppressor of cytokine signaling; STAT3, signal transducer and activator of transcription-3; TRAF6, TNF receptor-associated factor-6 [Figure modified according to Straub (121) and Reich (137)].

Based on the considerations mentioned above, it seems reasonable that the proinflammatory cytokine IL-17 interferes with insulin signaling and insulin sensitivity (Figure 2) and contributes to adipoflammation and metaflammation in obesity and diabetes (139). Since IL-17 activates the IκB kinase (IKK)/NFκB pathway, inhibitory phosphorylation of IRS-1 directly by IKK and indirectly by JNK activation in response to other proinflammatory cytokines is able to attenuate insulin sensitivity (140) on a molecular basis. Moreover, classical proinflammatory pathways in adipocytes such as TNF- and LPS-induced IL-6 and CCL20 production are augmented by IL-17 (141).

Interestingly, IL-17A/IL-17A receptor signaling can promote adipogenic transdifferentiation of myoblast cells via activation of the main adipogenic transcription factor PPARγ (142). In contrast, in human mesenchymal stem cells and in adipocytes, IL-17 inhibits adipocyte differentiation (143, 144) and the expression of adipokines and adipogenic transcription factors. In differentiated adipocytes, IL-17 upregulates lipolysis and IL-6/IL-8 secretion (143), whereas it inhibits the expression of adipogenic transcription factors and the cellular uptake of glucose (127). Most importantly, IL-17-deficient mice are characterized by enhanced insulin sensitivity and increased glucose uptake (127). In human co-culture experiments, macrophage-derived IL-1β was shown to enhance IL-17 production (145). Since macrophages do express IL-17 receptors, there might exist a positive and paracrine feedback loop that enhances local visceral adipose tissue inflammation. Importantly, in KK-Ay diabetic mice, antibody-mediated neutralization of elevated IL-17 concentrations improved insulin resistance and increased glucose uptake by skeletal muscle, but not by adipose tissue (146). Additionally, the expression of adipogenic differentiation markers and adiponectin was upregulated.

Most interestingly, IL-17, adipose tissue inflammation, and psoriasis might be directly connected on a molecular basis. Adipocytes are able to synthesize and to secrete significant quantities of VEGF (vascular endothelial growth factor) that can be further enhanced in the context of hyperinsulinemia. Since VEGF induces keratinocyte inflammation by an interaction with IL-17, hyperinsulinemia in the context of visceral obesity and/or type 2 (pre)-diabetes might further increase severity or susceptibility of psoriasis (147, 148).

### Potential Therapeutic Implications of Anti-IL-17 in Metabolic Diseases

From an evolutionary point of view, immune system, and metabolism are connected and cross-regulated by common proinflammatory molecules. Based on epidemiological data, the inflammatory state in severe psoriasis is positively associated with obesity and type 2 diabetes. IL-17, one of the main and causative proinflammatory cytokines in psoriasis, mechanistically links inflammation with insulin resistance and adipocyte dysfunction (127, 143, 144). In contrast, neutralization of IL-17 improves insulin sensitivity and glucose uptake in animal studies (130, 146). Considering these data, it is intriguing to speculate whether long-term neutralization of IL-17 in humans improves co-existent type 2 diabetes or even reduces the risk of diabetes manifestation. This hypothesis has to be investigated by randomized, placebo-controlled, clinical long-term studies in patients with poorly controlled type 2 diabetes and in pre-diabetic patients, respectively. With respect to available *in vitro* data and to data from experimental animal studies, neutralization of IL-17 should rather improve a coexistent type 2 diabetes than worsen it (127, 146). In addition, biologicals targeting the IL-17 or IL-12/23 pathway seem to be weight-neutral and do not worsen preexistent type 2 diabetes or increase the risk of diabetes manifestation (148–155).

## Future Clinical Perspectives

**Figure 3:**
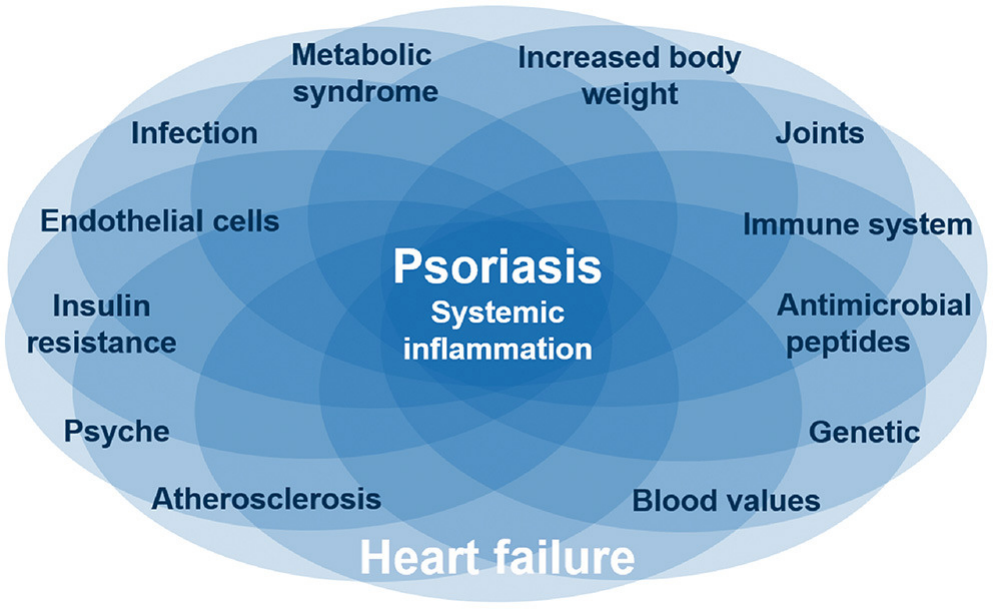
Common pathways for psoriasis, cardiovascular disease, and metabolic syndrome. Several diseases like psoriasis and heart failure are based on systematic inflammation, which is linked by IL-17, one of the main and causative proinflammatory cytokines in psoriasis. These inflammatory diseases are associated with certain interacting factors like obesity and depression. The complex interaction between inflammatory diseases and different factors is reflected by individual organ manifestations of the same inflammatory disease. Nevertheless, it is still unclear which factors are originating from the inflammation or are causing the inflammation.

Psoriasis skin manifestations, cardiovascular as well as metabolic disease in psoriasis appear to share pathogenic mechanisms evolving around IL-17 and its proinflammatory role (Figure 3). First evidence suggests that anti-IL-17A therapy not only improves skin manifestations of psoriasis, but also cardiovascular inflammation as well as (potentially) metabolic factors. If other anti-inflammatory drugs (e.g., biologics, immunosuppressants) have similar effects is currently not known.

What do we recommend to our patients with moderate to severe psoriasis based on these findings? First, thorough screening for subclinical cardiovascular diseases should be performed, including assessment of additional cardiovascular risk factors to help minimize the potential risk of developing cardiovascular events. Since severe psoriasis with a high inflammatory burden is associated with increased cardiovascular problems, effective control of skin inflammation is critical. In addition, signs for existing cardiovascular or metabolic disease need special attention and treatment (if required). Second, based on the findings discussed in the present review, the existence of systemic comorbidity (psoriasis arthritis, cardiovascular disease, metabolic disease) should trigger usage of anti-inflammatory systemic treatment. Among the various options, it appears that the best evidence so far is present for an effect of anti-IL-17. Controlled studies addressing this, however, are missing.

In summary, a picture emerges that underlines the complex interplay of several or even multiple cytokines or isoforms thereof in the context of psoriasis and its comorbidities. Clearly, synergies exist resulting in increased inflammation when two cytokines jointly affect a given target cell, e.g., with regard to IL-8 production of synoviocytes or skin fibroblasts stimulated with TNF-α plus IL-17A or -F (156). This opens up an opportunity for a more “complete” normalization of an inflamed target tissue through blockade of more than one cytokine, as again exemplified by the more complete normalization of the gene expression profile of fibroblasts stimulated with the supernatant of Th17 cells through simultaneous blockade of IL-17A and -F in comparison to blocking either one (156).

Further evaluation of the therapeutic potential of strategies aimed to blocking more than one IL-17 isoform would substantially benefit from a deepened understanding of the synergies amongst these isoforms as well as their quite different and sometimes even opposite functions in a tissue-specific manner (17).

## Author Contributions

All authors contributed equally to conception and writing of the paper. All authors contributed to manuscript revision, read, and approved the submitted version.

### Conflict of Interest

ES received honoraria as a speaker or advisor from Novartis, Miltenyi, and Celgene. She received a research grant to study therapeutic modulation of regulatory T cells and myeloid cells in psoriasis patients from Novartis. W-HB received honoraria as a speaker or advisor from Abbvie, Almirall, BMC, Celgene, Janssen, Leo, Lilly, Novartis, UCB. He received a research grant to study effects of to facitinib on monocytes and macrophages from Pfizer. KG has been a consultant, lecturer, or investigator for AbbVie, Almirall, Biogen, Boehringer Ingelheim, Brisol-Myers Squibb, Celgene, Eli Lilly, Janssen-Cilag, MSD Sharp & Dohme, Novartis, Pfizer, Roche, UCB Pharma. TG has received honoraria as a speaker or advisor from several pharmaceutical and medical device companies including Abbott Vascular, St Jude medicals, Neovasc, Bayer, Novartis. ZK has received an honorarium as an advisor from Novartis. DT has been a consultant and advisor and/or received speaking fees and/or grants and/or served as an investigator in clinical trials for the following companies: AbbVie, Almirall, Amgen, Bioskin, Boehringer Ingelheim, BMS, Celgene, Dignity, Eli Lilly, Galapagos, GSK, LEO Pharma, Janssen-Cilag, MSD, Morphosis, Novartis, Pfizer, Regeneron, Roche, Sandoz, Sanofi, and UCB. AS has received an honorarium as an advisor from Novartis.
